# Reply to Ionescu, E.C. Comment on “Chen et al. Vestibulo-Ocular Reflex Results in Patients with Intralabyrinthine Schwannomas: Case Series with a Literature Review. *Diagnostics* 2025, *15*, 2093”

**DOI:** 10.3390/diagnostics16101538

**Published:** 2026-05-19

**Authors:** Xiaoye Chen, Yingzhao Liu, Yangming Leng, Ping Lei, Xingqian Shen, Kaijun Xia, Qin Liu, Ziying Xu, Bo Liu, Hongjun Xiao

**Affiliations:** 1 Department of Otorhinolaryngology-Head and Neck Surgery, ENT Institute, Union Hospital, Tongji Medical College, Huazhong University of Science and Technology, Wuhan 430022, China; 2 Hubei Province Clinical Research Center for Deafness and Vertigo, Wuhan 430022, China; 3 Department of Radiology, Union Hospital, Tongji Medical College, Huazhong University of Science and Technology, Wuhan 430022, China

We sincerely thank the Editor for the opportunity to respond to this correspondence and are grateful to the authors for their thoughtful and constructive comments on our article [1]. We appreciate their careful reading of our work and their valuable suggestions regarding internal auditory canal (IAC) morphology, symptom attribution, and additional vestibular assessments. These comments help refine the interpretation of our findings and provide important directions for future studies on intralabyrinthine schwannoma (ILS).

**Comment 1:**

**Reply:** We thank the authors for this insightful comment regarding IAC morphology. We agree that reduced IAC dimensions may represent a relevant anatomical factor in patients with cochleovestibular symptoms, and that dedicated coronal assessment may improve diagnostic precision. We would also like to clarify that our interpretation was primarily based on the classical literature, in which narrowed IAC is a computed tomography (CT)/ high-resolution CT-based radiological entity, generally defined by an axial or mid-point IAC diameter of less than 2 mm, with magnetic resonance imaging (MRI) mainly used to assess cochleovestibular nerve morphology rather than to define classical narrowed IAC by size [2].

In response to this comment, we re-reviewed the original MRI data with our radiologist (Figure 1). The coronal IAC diameters were 3.73 mm and 3.63 mm in Cases 2 (Figure 1(2A)) and 4 (Figure 1(4A)), respectively, which did not support narrowed or near-narrowed IAC. In Case 3, the coronal IAC diameter was 2.42 mm, with an axial diameter of 3.25 mm (Figure 1(3A)), and this case may therefore meet the more recently proposed morphometric concept of near-narrowed IAC on coronal MRI. We consider this newer concept to be a useful anatomical refinement rather than a contradiction of the classical definition. However, the original narrowed/near-narrowed IAC literature describes this entity primarily as a vestibular-paroxysmia-like condition characterized by brief recurrent vertigo, while auditory symptoms may coexist but are not its defining clinical pattern [3,4,5].

Accordingly, although IAC morphology may have acted as a contributing anatomical modifier in Case 3, the overall clinical–radiological pattern was not fully explained by near-narrowed IAC alone. This patient had a 30-year history of chronic progressive cochlear symptoms including hearing loss and tinnitus and recurrent vertigo episodes lasting several minutes to hours. Vestibular testing revealed impaired superior vestibular nerve function demonstrated as ipsilateral caloric hypofunction and absent ocular vestibular evoked myogenic potential (oVEMP). Her MRI findings included an intravestibulocochlear schwannoma and decreased signal intensity in the ipsilateral semicircular canal, which corresponded to the dysfunction of the cochlear and superior vestibular nerve-related pathway. Taken together, these findings favor ILS-related labyrinthine involvement as the principal explanation, with IAC morphology considered a potential modifier rather than an alternative diagnosis. We appreciate this valuable suggestion and agree that future studies should systematically include dedicated IAC morphometry, especially on coronal images, to further refine symptom attribution in complex cases.

**Comment 2:**

**Reply:** We thank the authors for this thoughtful and clinically important comment. We agree that vestibular symptoms in patients with ILS should be interpreted with caution, particularly because ILS has a heterogeneous clinical spectrum and may overlap with Ménière-like audiovestibular syndromes and other retrocochlear disorders. We also agree that recurrent vestibular attacks should not be regarded as the predominant phenotype of all ILS cases.

In our article, we did not intend to suggest that vestibular symptoms are the dominant manifestation across the entire ILS population. In fact, our literature review showed that auditory symptoms were more frequent than vestibular symptoms: among 150 patients with detailed audiovestibular symptomatology, 141 patients presented with hearing loss and 101 with tinnitus, whereas vestibular symptoms were documented in 84 patients. However, only four cases in our case series and all four patients had vestibule-involving lesions, including two intravestibular and two intravestibulocochlear schwannomas, which may explain the relatively prominent vestibular phenotype in our cohort. Moreover, our interpretation was not based solely on subjective vestibular symptoms, but also on objective vestibular findings, including unilateral caloric hypofunction in all four patients and abnormal video head impulse test (vHIT) results in two of the three patients who underwent vHIT.

Thus, our results actually reflect the vestibular-dominant phenotype in vestibule-involving ILS rather than in all ILS. We fully agree that vestibular symptoms in these patients may be influenced by multiple factors, including tumor location, labyrinthine microenvironmental changes, and potentially other anatomical or retrocochlear mechanisms. This is also consistent with our conclusion that impaired vestibulo-ocular reflex (VOR) function in ILS may result not only from anatomical disruption but also from biochemical or metabolic alterations within the inner ear. We appreciate the authors’ comment, which helps refine the interpretation of symptom attribution in ILS and highlights the need for future studies with larger cohorts, subtype-stratified analyses, and comprehensive anatomical and vestibular assessments.

**Comment 3:**

**Reply:** We thank the authors for this valuable methodological comment. We agree that the hyperventilation test may provide useful complementary information in patients with suspected retrocochlear or eighth cranial nerve dysfunction. After reviewing the four studies cited by the authors, we found that they generally support the view that hyperventilation may reveal latent unilateral vestibular asymmetry or eighth cranial nerve involvement and may therefore have some adjunctive value in vestibular schwannoma [6,7,8,9]. At the same time, the currently available evidence remains limited, as two of the cited reports were case-based [6,7] and the two case–control studies showed moderate-to-high sensitivity with variable specificity depending on the comparator group [8,9].

In our study, hyperventilation testing was not included in the routine vestibular assessment protocol, which included caloric testing, vHIT, cervical vestibular evoked myogenic potential (cVEMP), and oVEMP. Also, hyperventilation-induced nystagmus is not specific for ILS and interpretating its results depends on the combination of clinical profile, functional and imaging findings. In this context, we believe that the hyperventilation test may serve as an optional adjunctive tool in revealing retrocochlear or local compressive mechanisms and not a substitute for caloric testing, vHIT, or MRI.

Therefore, the absence of hyperventilation test did not change the interpretation and conclusion of our study, which was based on MRI findings and objective vestibular abnormalities. We are grateful for this suggestion and agree that future studies may usefully consider including hyperventilation testing as an adjunctive assessment of possible retrocochlear involvement.

We again sincerely thank the Editor for the opportunity to address these comments and are grateful to the authors for their careful reading and constructive suggestions. Their comments have helped us clarify the interpretation of our cases and further improve the discussion of anatomical, functional, and radiological factors in ILS. We appreciate this valuable academic exchange and believe it contributes to a more comprehensive understanding of vestibular dysfunction in this rare condition.

## Figures and Tables

**Figure 1:**
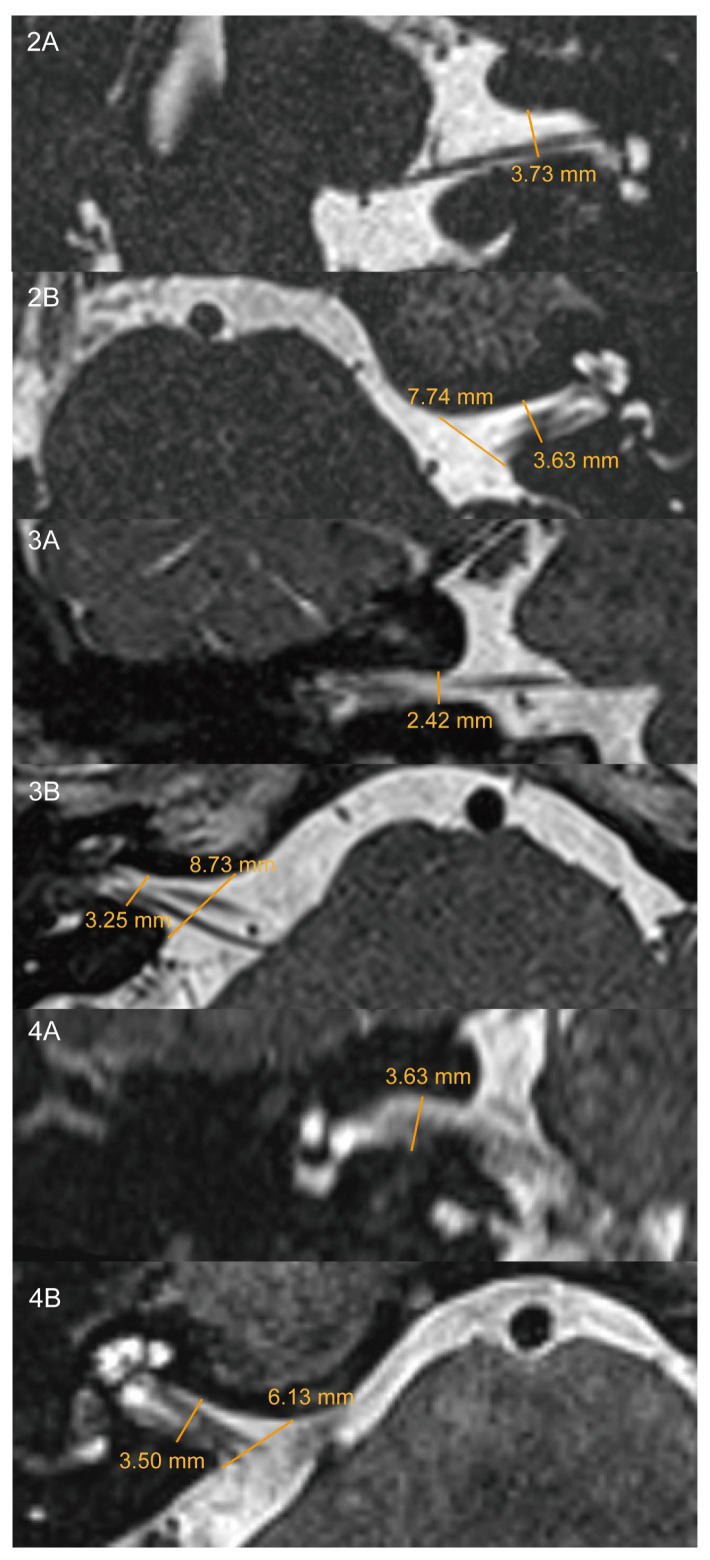
Coronal and axial high-resolution T2-weighted MRI images showing IAC measurements in Cases 2–4. Coronal images (**2A**,**3A**,**4A**) demonstrate IAC widths of 3.73 mm, 2.42 mm, and 3.63 mm, respectively. Axial images (**2B**,**3B**,**4B**) show corresponding axis widths of 3.63 mm, 3.25 mm, and 3.50 mm, respectively. IAC, internal auditory canal.
